# Inducible *Ift88*‐deficient mice show features consistent with mild pulmonary hypertension

**DOI:** 10.14814/phy2.70749

**Published:** 2026-01-26

**Authors:** Selina M. Garcia, Benjamin J. Lantz, Helen J. Wagner, David T. Jones, Rene Arechiga‐Gonzalez, Tamara A. Howard, Sana Gul, Terry H. Wu, Thomas F. Byrd, Olivia C. Heath, Laura V. Gonzalez Bosc

**Affiliations:** ^1^ Cell Biology and Physiology Department The University of New Mexico Health Sciences Center Albuquerque New Mexico USA; ^2^ Center for Infectious Disease and Immunity The University of New Mexico Health Sciences Center Albuquerque New Mexico USA; ^3^ Department of Internal Medicine The University of New Mexico Health Sciences Center Albuquerque New Mexico USA; ^4^ Biochemistry and Molecular Biology Department The University of New Mexico Health Sciences Center Albuquerque New Mexico USA

**Keywords:** arterial remodeling, cilia, Ift88, polaris, pulmonary hypertension

## Abstract

Intraflagellar transport protein 88 (IFT88) is essential for primary and motile cilia formation. In murine models and humans, Ift88 mutations contribute to renal cysts, epithelial proliferation and impaired immune responses. In mice, Ift88 knockout (KO) reduces airway cilia, increases airway epithelial proliferation and hyperreactivity, elevates IL‐22 and decreases lung T regulatory cells. Pulmonary hypertension (PH) is a deadly disease marked by aberrant metabolism and immunoinflammatory mediators causing vasoconstriction and vascular remodeling. Endothelial‐to‐mesenchymal transition (EndMT) contributes to PH, and endothelial‐specific Ift88 KO promotes endothelial proliferation and EndMT. We hypothesized that global loss of Ift88 causes PH. We assessed PH indices at 2 and 14 weeks postdeletion in tamoxifen‐inducible Ift88 KO mice. These mice showed signs of PH, including increased right ventricular systolic pressure, cell proliferation in the walls of resistance arteries, and arterial wall thickening. At the early time point examined, we did not detect evidence of lung inflammation or EndMT. Because this is a tamoxifen‐induced global Ift88 KO model, we cannot attribute the PH features to gene deletion in specific vascular cells, nor can we rule out the possibility that tamoxifen administration, global Ift88 deletion, the associated weight gain and food restriction may have influenced cardiovascular physiology in these mice.

## 1 INTRODUCTION

IFT88 is an intraflagellar transport (IFT) protein required for primary and motile cilia formation (Gilley et al., 2014). Primary cilia are organelles characterized by small antenna‐like cellular protrusions that detect and transduce chemical and mechanical cues from the extracellular environment to maintain cell and tissue development and homeostasis. Primary cilium and the IFT system form a crucial signaling hub whose structure and/or function are disrupted in a group of human disorders collectively known as ciliopathies (Wang et al., 2021). Additionally, the IFT system regulates the polarized transport of endosome‐associated T‐cell receptors and signaling intermediaries during the assembly of the immune synapse in lymphocytes, which lack a primary cilium (Finetti et al., 2011).

Mutations in the *IFT88* gene are known to occur in humans (McIntyre et al., 2012). Deletion of or mutations in the *IFT88* gene lead to epithelial proliferation at multiple sites in the body (McIntyre et al., 2012; Richards et al., 1996; Robert et al., 2007). For example, mice develop renal cysts after 6 months of inducible *Ift88* gene knockout (KO) due to epithelial proliferation (Gilley et al., 2014). A marked reduction in cilia in respiratory epithelia after 14 weeks of *Ift88* gene deletion led to airway epithelia proliferation (Nava et al., 2022). Furthermore, loss of airway cilia resulted in noninflammatory bronchiectasis and hyperreactive airways (Gilley et al., 2014). Despite the absence of overt signs of inflammation, Nava et al. showed that these mice had significantly elevated IL‐22 levels and a decreased percentage of T regulatory cells (Tregs) in bronchoalveolar lavage fluid (BALF) after 14 weeks of *Ift88* gene deletion. Furthermore, *Ift88* KO mice develop a persistent lung infection when inoculated with *Mycobacterium abscessus* (*M. abscessus*)‐embedded agarose beads, whereas control mice clear the infection from their lungs (Nava et al., 2022). These results are consistent with the multifaceted role motile‐ciliated cells of the respiratory tract play in pulmonary innate immune defenses in addition to mucociliary clearance (Whitsett & Alenghat, 2015).

As expected, a loss of airway cilia reduces mucociliary clearance, leading to mucus buildup and stasis, and potentially bronchiectasis. Interestingly, 48% of patients with noncystic fibrosis bronchiectasis present with pulmonary hypertension (PH), with more severe lung airway disease (Goeminne et al., 2012). PH is defined as a mean pulmonary artery pressure (PAP) >20 mmHg under resting conditions (normal 12–14 mmHg) (Simonneau et al., 2019). PH causes right ventricular hypertrophy and leads to right‐sided heart failure. The World Health Organization has established five classifications of PH. Group 1 or Pulmonary Arterial Hypertension (PAH) is a rare disease that can be idiopathic, familial due to mutations in the bone morphogenetic protein receptor type II (BMPR2) gene, caused by toxins (amphetamines, cocaine), connective tissue diseases, human immunodeficiency virus (HIV), congenital heart diseases, etc. Group 2 is pulmonary venous hypertension due to left‐sided heart disease or left‐sided valvular defects, and it is the most common type of PH. Group 3 is the second most common and is associated with hypoxemia (i.e., chronic hypoxia), typically secondary to chronic lung diseases such as chronic obstructive pulmonary disease (COPD), bronchiectasis, interstitial lung diseases or residence at high altitudes. Group 4 includes chronic thromboembolic PH, and group 5 is considered multifactorial due to tumors and other causes (Simonneau et al., 2019).

PH is a progressively debilitating condition characterized by aberrant cellular, metabolic and molecular pathways within the pulmonary arteries (Jernigan & Resta, 2013; Stenmark et al., 2006; Weise‐Cross et al., 2019; Yan et al., 2020) and immunoinflammatory mediators (Hamidi et al., 2008; Hosokawa et al., 2013; Price et al., 2012; Wang et al., 2011) that enhance vasoconstriction and cause vascular remodeling. Furthermore, endothelial‐to‐mesenchymal transition (EndMT) plays a crucial role in the pathogenesis of PH (Gorelova et al., 2021). Interestingly, the loss of primary cilia from vascular endothelial cells (EC) in mice with EC‐specific *Ift88* gene deletion enhances pulmonary EC proliferation and promotes EndMT (Singh et al., 2020). However, PH was not assessed in those mice. Furthermore, a loss of cilia elongation upon cytokine stimulation is associated with endothelial dysfunction in patients with PAH (Dummer et al., 2018). Therefore, we hypothesized that global loss of the *Ift88* gene leads to the development of PH. The main findings of this study are that inducible‐*Ift88* global KO mice show signs of mild PH as early as 2 weeks postgene deletion, associated with increased pulmonary arterial wall cell proliferation, but no significant lung inflammation or signs of EndMT.

## 2 METHODS

### 2.1 Animal model

All experimental procedures were approved by the Institutional Animal Care and Use Committee (IACUC) of the University of New Mexico Health Sciences Center. Animals were randomly assigned to groups, and investigators were blinded to treatment protocols. Mice were multihoused in Lab Products Super Mouse 1800 microisolator cages and had access to standard rodent diet (5V5R from LabDiet) and water ad libitum using hydropac water pouches (unless specified). All mice used for experiments were euthanized by exsanguination and removal of the lung and heart following anesthesia with 2.5–3% isoflurane in 100% oxygen.

The mouse model employed in these studies was selected based on prior findings showing that conditional deletion of the *Ift88* gene via the Cre/loxP system leads to spontaneous development of bronchiectasis in adult mice, without the need for bacterial infection (Gilley et al., 2014). This model features a conditional mutation in *Ift88*, a gene crucial for the formation and function of normal cilia. In mice homozygous for the floxed *Ift88* allele and expressing Cre recombinase, tamoxifen treatment resulted in approximately an 80% reduction in IFT88 protein within 2–3 weeks, accompanied by the progressive emergence of bronchiectasis around week three postdeletion. Conversely, mice lacking Cre recombinase exhibited wild‐type IFT88 expression following tamoxifen administration.

To achieve gene deletion, *Ift88* floxed mice (B6.129P2‐Ift88^tm1Bky^/J, Jackson Laboratory) were bred with mice harboring a tamoxifen‐inducible Cre recombinase under an actin promoter (B6.Cg‐Tg(CAG‐Cre/Esr1*)5Amc/J, Jackson Laboratory). Genotyping was performed at ~28 days of age using PCR (Transnetyx; Cordova, TN or in our laboratory using XNAT‐100RXN kit from Sigma) to identify the presence of floxed *Ift88* (FWD: 5′‐GCGGCTGCAGAGATCCA‐3′; REV: 5′‐GGTATTGTTAGGAAGTAGTAAAACATAA‐3′), wild‐type *Ift88* (FWD: 5′‐AGTAAGCGGCTGCAGAGATC‐3′; REV: 5′‐AATTCTGGCTCTGAACACAATCC‐3′) and Cre sequences (FWD: 5′‐TTAATCCATATTGGCAGAACGAAAACG‐3′; REV: 5′‐CAGGCTAAGTGCCTTCTCTACA‐3′). Male and female mice positive for both Cre and floxed *Ift88* alleles formed the experimental group (*Ift88* KO or Cre+); those negative for Cre served as controls. Both groups received tamoxifen (Sigma‐Aldrich; 2 mg/100 μL in corn oil; ~80/mg/kg) via daily intraperitoneal injections over 5 days. As all mice were exposed to tamoxifen, observed differences are attributable to *Ift88* gene deletion rather than the drug itself. Moreover, the short treatment window (5 days) relative to the ~2‐week to 3‐month experimental timeline reduces the likelihood of a sustained tamoxifen effect.

Beyond bronchiectasis, *Ift88*‐deficient mice develop renal cysts approximately 6 months postinduction, which falls outside the scope of this study. The control group had an average age of 169 days ± 3 days, and the Cre + group had an average of 166 days ± 5 days (*p* < 0.05).

Additionally, IFT88 loss has been associated with altered feeding behavior and subsequent weight gain, likely due to disrupted hypothalamic cilia involved in appetite regulation—a phenotype that we observed in Cre‐positive *Ift88* floxed mice after tamoxifen treatment. Food intake monitoring revealed that age‐matched Cre‐negative *Ift88* floxed mice consumed ~2.0 g of standard chow (Taklad 2920X; Envigo) daily. To ensure consistent dietary conditions, this quantity was provided to all mice throughout the study.

### 2.2 Indices of pulmonary hypertension

Peak right ventricular systolic pressure (RVSP), a surrogate for pulmonary artery pressure, was measured in isoflurane (~2.5%/100% O_2_)‐anesthetized mice via a closed‐chest transdiaphragmatic approach, as previously described (Maston et al., 2017; Ramiro‐Diaz et al., 2013; Sheak et al., 2020). Briefly, the diaphragm was exposed through an upper transverse laparotomy, and a 25‐gauge needle connected to a pressure transducer (P23 XL; Spectramed, Columbus, OH) was inserted into the right ventricle (RV) of the heart. The signal was amplified using a Gould Universal amplifier. RVSP and heart rate were recorded using a computer‐based data acquisition system (AT‐CODAS, DATAQ Instruments, Akron, OH). Following data collection, the heart was excised, and the atria, fat and major vessels were removed. RV hypertrophy (Fulton's index) was calculated as the ratio of RV weight to the combined weight of the left ventricle and septum (LV + S). Body weight (BW) was also measured. Blood samples were obtained via direct cardiac puncture with a heparinized capillary tube, then centrifuged, and the % hematocrit was calculated as the ratio of red blood cells to the total sample volume.

### 2.3 Immunofluorescence

Following the acquisition of hemodynamic data, lungs were perfused through the right ventricle with ~5 mL of a modified HEPES‐buffered physiological saline solution (134 mM NaCl, 6 mM KCl, 1 mM MgCl, 10 mM HEPES, 2 mM CaCl_2_, 0.026 mM EDTA and 10 mM glucose) containing heparin, 4% albumin (Sigma) and 10^−4^ M papaverine (Sigma) at a pressure of 20 mmHg. The heart, lungs and trachea were removed. All salts were purchased from Sigma‐Aldrich (St. Louis, MO). The lungs were inflated through the trachea with the same solution. Half of the lung was fixed in 4% paraformaldehyde (Polyscience, Warrington, PA) in phosphate‐buffered saline (PBS) and embedded in paraffin. The other half was fixed in 2% formaldehyde in PBS, followed by cryoprotection in 20% sucrose, and embedding in optimal cutting temperature (OCT) compound.

Pulmonary arterial remodeling was evaluated using a previously published method (de Frutos et al., 2007). Paraffin‐embedded lung tissue sections (5 μm thickness) were deparaffinized and rehydrated, followed by a 20‐minute antigen retrieval step at ≥ 90°C using a buffer composed of 10 mM Tris (pH 9.0) and 1 mM EDTA, using a rice cooker. The sections were then blocked and permeabilized in a solution containing 1× PBS, 2% normal donkey serum and 0.1% Triton X‐100. Lung sections were stained with a rabbit anti‐smooth muscle α‐actin (SMA) antibody (1:400, NB600‐531 Novus). Detection was carried out using DyLight 549‐conjugated donkey anti‐rabbit secondary antibody (1:350, Thermo Fisher Scientific). Sections were counterstained with SYTOX Green (1:10,000, S7020 ThermoFisher) and Alexa Fluor 633 hydrazide (0.05 μM, A30634 ThermoFisher) to label nuclei and the elastic lamina, respectively. Images were acquired using a 63× objective with a Leica TCS SP5 Spectral Confocal System (Buffalo Grove, IL) and LAS AF 2.7.3.9723 software. Vessels sectioned at nonperpendicular angles were excluded from analysis. For each animal, approximately 10 arteries with an outer diameter < 60 μm were assessed. To determine arterial wall thickness, an AI‐based classifier was trained to recognize SMA‐positive staining specific to arterial structures, using HALO software (version 4.1, Indica Labs). The classifier‐to‐annotations feature was then used to automatically generate annotations around the external and internal boundaries of identified arteries, creating positive and negative annotations, respectively. Percent wall thickness was calculated using the formula: (external diameter−luminal diameter)/(external diameter) × 100. Diameters were derived from the circumferences of the external and internal annotations. Classifier parameters and thresholding were optimized for each analysis to ensure reproducibility and accuracy across all samples. SMA fluorescence intensity was also quantified and reported as % SMA+ area. HALO analysis was validated by comparing it with manual selection of internal and external arterial borders in ImageJ, as previously reported (Bierer et al., 2011; Maston et al., 2017).

For the detection of the self‐antigen collagen V, frozen lung tissue sections (~10 μm) were permeabilized using 0.1% Triton X‐100 and subsequently blocked with 5% normal donkey serum (017–000‐121 Jackson Immunoresearch) in phosphate‐buffered saline (PBS). The sections were then incubated with 0.1% gelatin (G2500 Sigma Aldrich) or a rabbit anti‐mouse collagen V (α1) primary antibody (1:400 in 0.1% gelatin), generously provided by Dr. David Birk (Department of Pathology and Cell Biology, University of South Florida College of Medicine), along with a goat anti–mouse SM22 Alpha/Transgelin antibody (1:250, ER‐14‐1416, RayBiotech; equivalent to Abcam ab10135). Detection was performed using DyLight 650‐conjugated donkey anti‐rabbit and DyLight 550‐conjugated donkey anti‐goat secondary antibodies (1:350, Thermo Fisher Scientific). Nuclei were counterstained with SYTOX Green (1:10,000, ThermoFisher). The anti‐collagen V antibody targets a peptide within exon 6, located downstream of the exon 3–4 region, and has been previously validated in Col5a1 knockout mice (Sun et al., 2011).

For quantifying TH17 cells, the same protocol used for SMA IF was applied. However, sections were incubated with rat monoclonal anti‐CD3 (1:100, ab11089, AbCam) and rabbit monoclonal anti‐RORγτ (1:3000, ab207082, AbCam) antibodies, followed by AlexaFluor 488 donkey‐anti‐rat (1:500, 712‐546‐153, Jackson) and DyLight 549 donkey‐anti‐rabbit (1:500, 711‐505‐152, Jackson), and counterstained with AlexaFluor 633 hydrazide (0.05 μM) to identify pulmonary arteries by their elastic lamina.

Imaging was carried out on a Leica TCS SP5 Spectral Confocal System. Using ImageJ (NIH), the medial and adventitial layers of pulmonary arteries were delineated. Software settings were adjusted to identify regions with collagen V signal, allowing for unbiased quantification of mean fluorescence intensity. Total CD3^+^ and CD3^+^ RORγτ^+^ cells present within the pulmonary arterial wall and perivascular region were manually counted. Pulmonary arteries were identified based on their morphological characteristics and positive staining for SM22 or elastin.

### 2.4 Immunohistochemistry

For assessing cell proliferation in the wall of pulmonary arteries, deparaffinized lung tissue sections following antigen retrieval, endogenous peroxidase quenching (0.5% H_2_O_2_), nonspecific epitope blocking (2% normal donkey serum, Jackson) and permeabilization (0.1% Triton X‐100) were incubated with a rabbit‐anti‐Ki67 SP6 clone antibody in 0.1% Tween 20 and 2% normal donkey serum in PBS (1:800; RM‐9106‐S0, ThermoFisher Scientific), followed by HRP‐donkey‐anti‐rabbit antibody (1:600, 711‐036‐152, Jackson Immuno Research).

For quantifying expression levels of EndMT markers and inflammation, lung tissue sections were processed as above and incubated with rabbit anti–smooth muscle α‐actin (SMA) antibody (1:400, NB600‐531, Novus), rabbit anti‐CD31 antibody (1:800, ab124432, AbCam), or goat anti‐IL‐22 (1:75, PA1‐21356, Invitrogen), followed by HRP‐donkey‐anti‐rabbit or goat (1:600, 711‐036‐152 or 705–036‐147, Jackson).

Sections were incubated with 0.5 mg/mL DAB (Sigma, D5905) + 0.025% H_2_O_2_ in PBS. All sections were counterstained with Mayer's hematoxylin (MHS16, Sigma), dehydrated and then coverslipped with CytoSeal.

Images were acquired using a slide scanner (Aperio AT2, Leica), and image analysis was performed using HALO image analysis software (version 4.1, Indica Labs). The Area Quantification module was used to quantify overall marker expression across the tissue section, employing a trained tissue classifier to distinguish between positive and nonpositive staining. For cellular proliferation analysis, the Cytonuclear module was used to quantify Ki‐67‐positive cells by detecting nuclear staining and calculating the labeling index. Classifier training and thresholding parameters were optimized for each marker to ensure consistency and accuracy across all slides. Measurements were performed either in the whole‐lung section or within manually annotated pulmonary arteries.

### 2.5 Statistical analysis

Results were expressed as the mean ± standard deviation (SD). Data were averaged and treated as a single observation for experiments with multiple measurements from an individual animal. Homoscedasticity and normality of residuals were assessed before selecting a parametric or nonparametric statistical test. Statistical significance was evaluated at the 95% confidence level (*p* < 0.05). A *t*‐test was used to compare two groups of continuous variables, while Fisher's Exact test was used to compare two groups of categorical variables. To determine the effect of sex, a two‐way ANOVA followed by Holm–Sidák multiple comparisons test was used.

Pearson correlations were computed among RVSP, body weight, sex (M = 0, F = 1) and genotype (Cre− = 0, Cre + = 1). A dendrogram was built using Ward.D2 hierarchical clustering on scaled variables; merge height indicates dissimilarity.

## 3 RESULTS

### 3.1 Features consistent with mild PH are present in mice with tamoxifen‐inducible global *Ift88* deletion

Prior research has demonstrated that deletion of the *Ift88* gene in ECs causes pulmonary vascular EC proliferation and EndMT (Singh et al., 2020), which are mechanisms implicated in the pathogenesis of PH (Gorelova et al., 2021). However, to our knowledge, the association between ciliopathy and PH has not been reported in animal models. Therefore, we tested the hypothesis that spontaneous PH develops in *Ift88* global KO mice, a mouse model previously characterized by our group. To test this hypothesis, we measured indices of PH in *Ift88* KO (Cre+) mice at 2 and 14 weeks after Ift88 gene deletion, compared with control (Cre−) mice. Two weeks was chosen as an early time point to ensure tamoxifen degradation (Valny et al., 2016), and the 14‐week time point was selected based on our prior publication demonstrating that Ift88 KO mice develop signs of bronchiectasis and lung inflammation (Nava et al., 2022).

**FIGURE 1:**
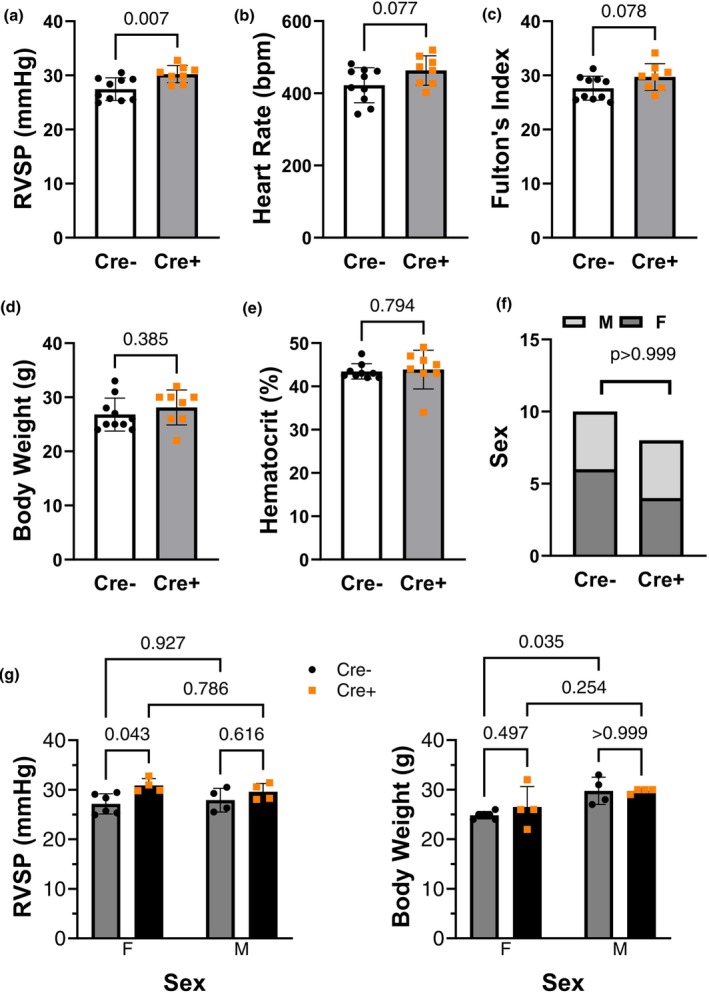
*Ift88* KO (Cre+) mice exhibit significantly elevated RVSP 2 weeks postgene deletion, suggesting early features of PH. (a) Right ventricular systolic pressure (RVSP), (b) heart rate, (c) Fulton's index (RV/LV + S), (d) body weight, (e) hematocrit and (f) sex distribution in *Ift88* KO (Cre+) and control (Cre−) mice 2 weeks after tamoxifen administration (*n* = 10 and 8, respectively). (g) Sex‐based analysis is also included (Cre− 6 F and 4 M, Cre + 4 F and 4 M). Data presented as mean ± SD—unpaired *t*‐test, Fisher Exact test and 2‐way ANOVA followed by Holm–Sidák multiple comparisons test, respectively.

Figure 1a shows that *IFT88* KO (Cre+) mice exhibit a significant increase in RVSP, a surrogate parameter of pulmonary arterial pressure, suggesting the presence of early features of PH. No difference was observed in Fulton's index, heart rate, hematocrit, body weight (BW) or sex distribution (Figure 1).

**FIGURE 2:**
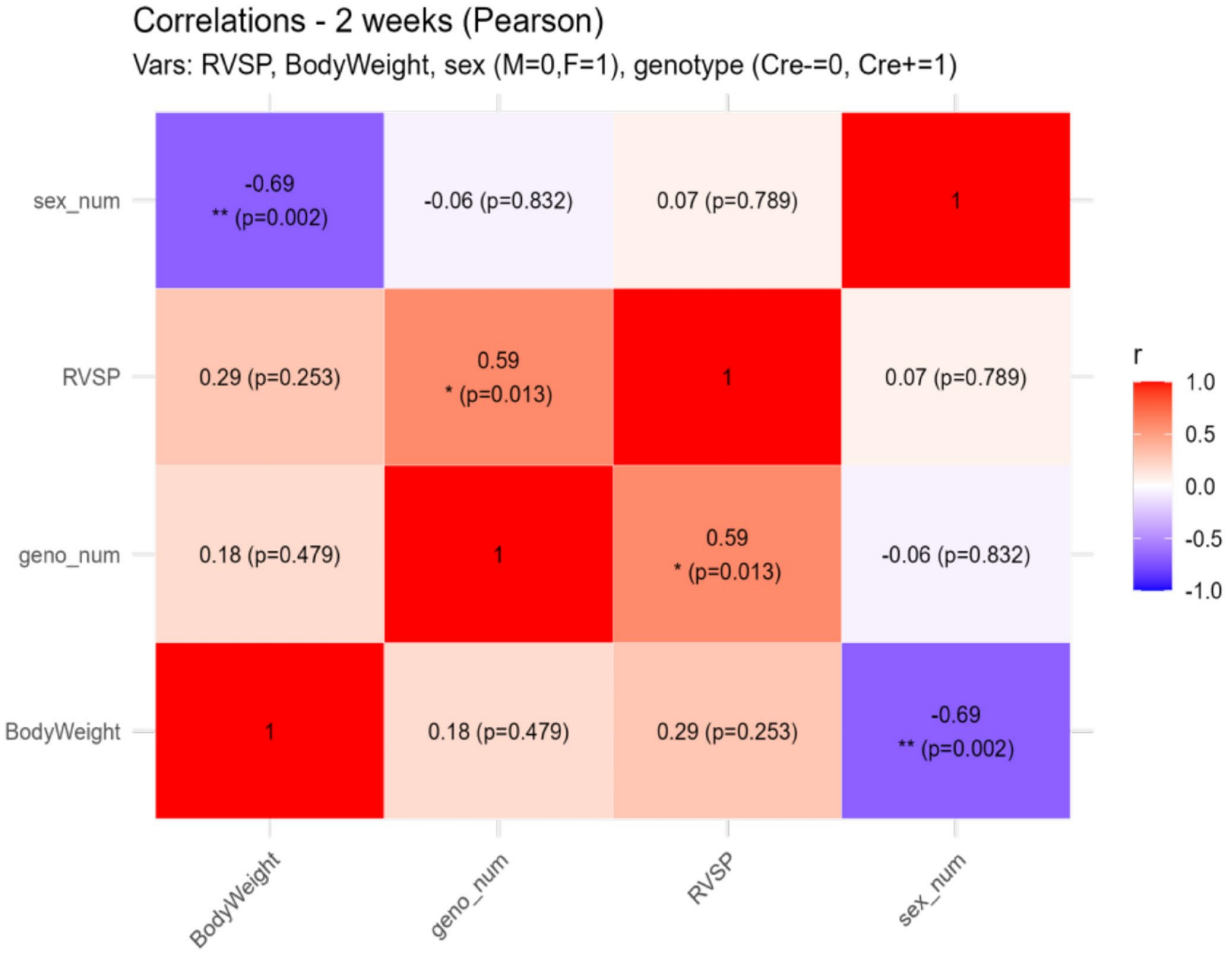
RVSP correlates with genotype in the two‐week cohort. Pearson correlations were computed among RVSP, body weight, sex (M = 0, F = 1) and genotype (Cre− = 0, Cre+ = 1). *p < 0.05, **p < 0.01, ***p < 0.001.

When RVSP was separated by sex, the data suggested a potential female bias, as RVSP was nearly significantly higher in Ift88 KO females when compared to control females (*p* < 0.0568), but similar to that in Ift88 KO males. When BW was separated by sex, control males had significantly higher BW than females (Figure 1g). This is further supported by a significant positive correlation between RVSP and genotype (Figure 2), whereas no additional correlations were observed with body weight or sex. However, body weight significantly correlated with sex. Increased pulmonary arterial wall thickness, a hallmark of PH, was absent between groups at this early time point (data not shown).

**FIGURE 3:**
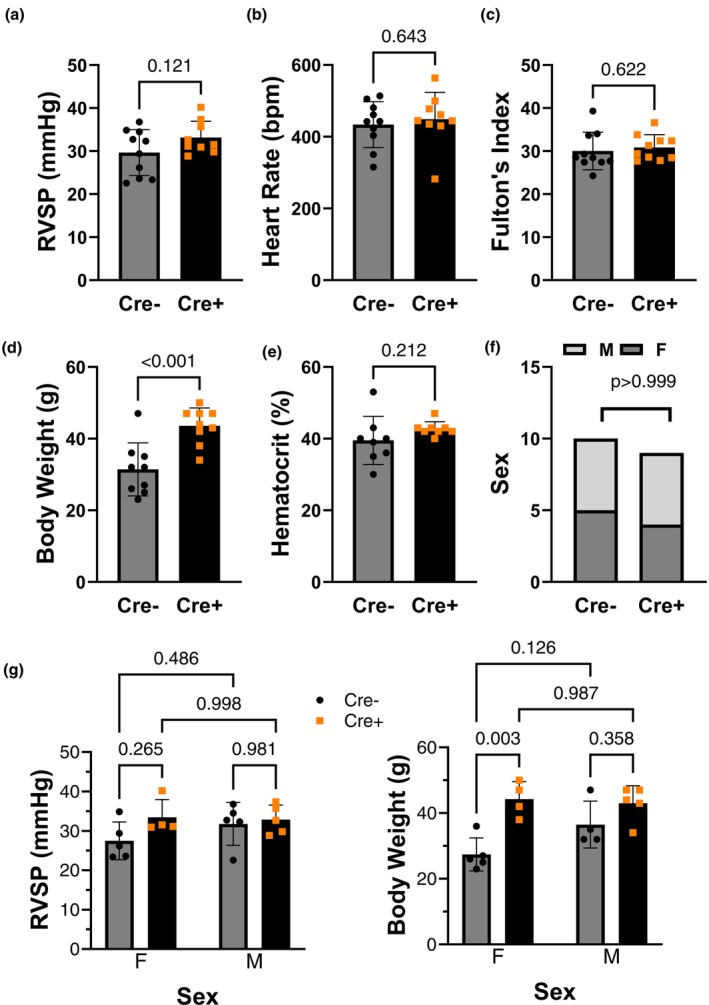
*Ift88* KO mice (Cre+) at 14 weeks post gene deletion no longer demonstrate increased RVSP. (a) Right ventricular systolic pressure (RVSP), (b) heart rate, (c) Fulton's index (RV/LV + S), (d) body weight, (e) hematocrit and (f) sex distribution in *Ift88* KO (Cre+) and control (Cre−) mice 14 weeks after tamoxifen administration. (*n* = 10 and 9, respectively). (g) Sex‐based analysis is also included. (Cre − 5 F and 5 M, Cre + 4 F and 5 M) Data presented as mean ± SD. Unpaired *t*‐test, Fisher Exact test and 2‐way ANOVA followed by Holm–Sidák multiple comparisons test, respectively.

After 14 weeks of *Ift88* gene deletion, when the *Ift88* KO mice had developed airway ciliopathy and remodeling, as well as lung inflammation (Nava et al., 2022), RVSP was no longer significantly different between groups (Figure 3a).

**FIGURE 4:**
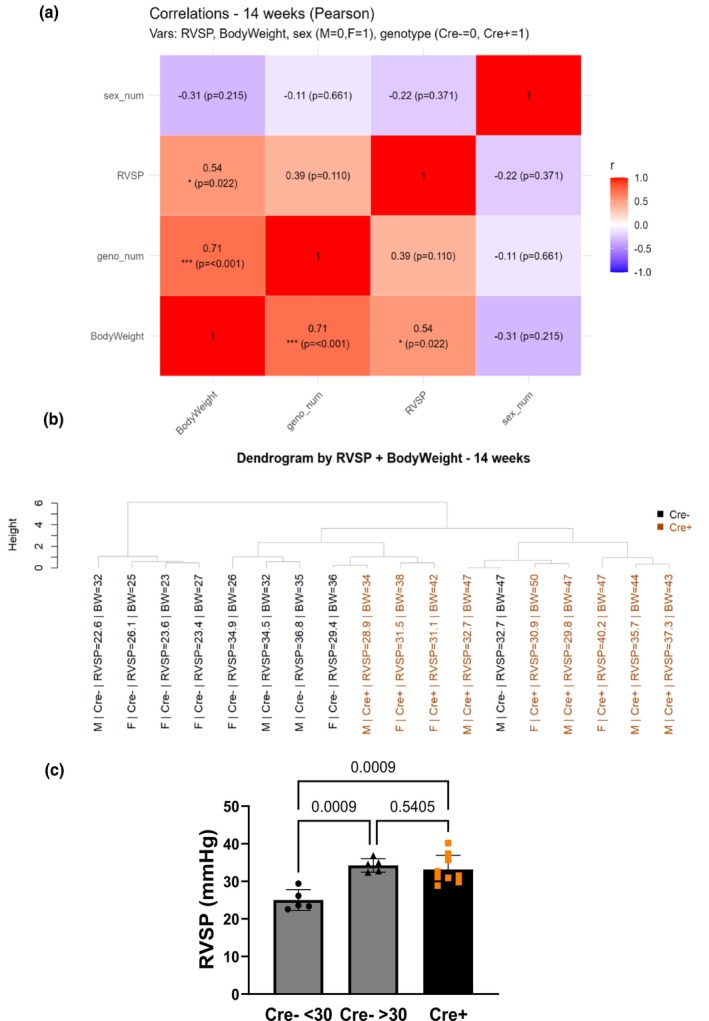
RVSP does not correlate with genotype, but with body weight in the 14‐week cohort. (a) Pearson correlations were computed among RVSP, body weight, sex (M = 0, F = 1) and genotype (Cre− = 0, Cre + =1). *p < 0.05, **p < 0.01, ***p < 0.001. (b) A dendrogram was built using Ward.D2 hierarchical clustering on scaled variables; merge height indicates dissimilarity. Big jumps indicate a natural cut. (c) RVSP among groups after separating the Cre‐group based on clustering results. Data presented as mean ± SD. One‐way ANOVA, followed by Holm–Sidák multiple comparisons test.

Separating the data by sex did not affect the results. RVSP showed a significant correlation with BW but not with genotype (Figure 4a).

**FIGURE 5:**
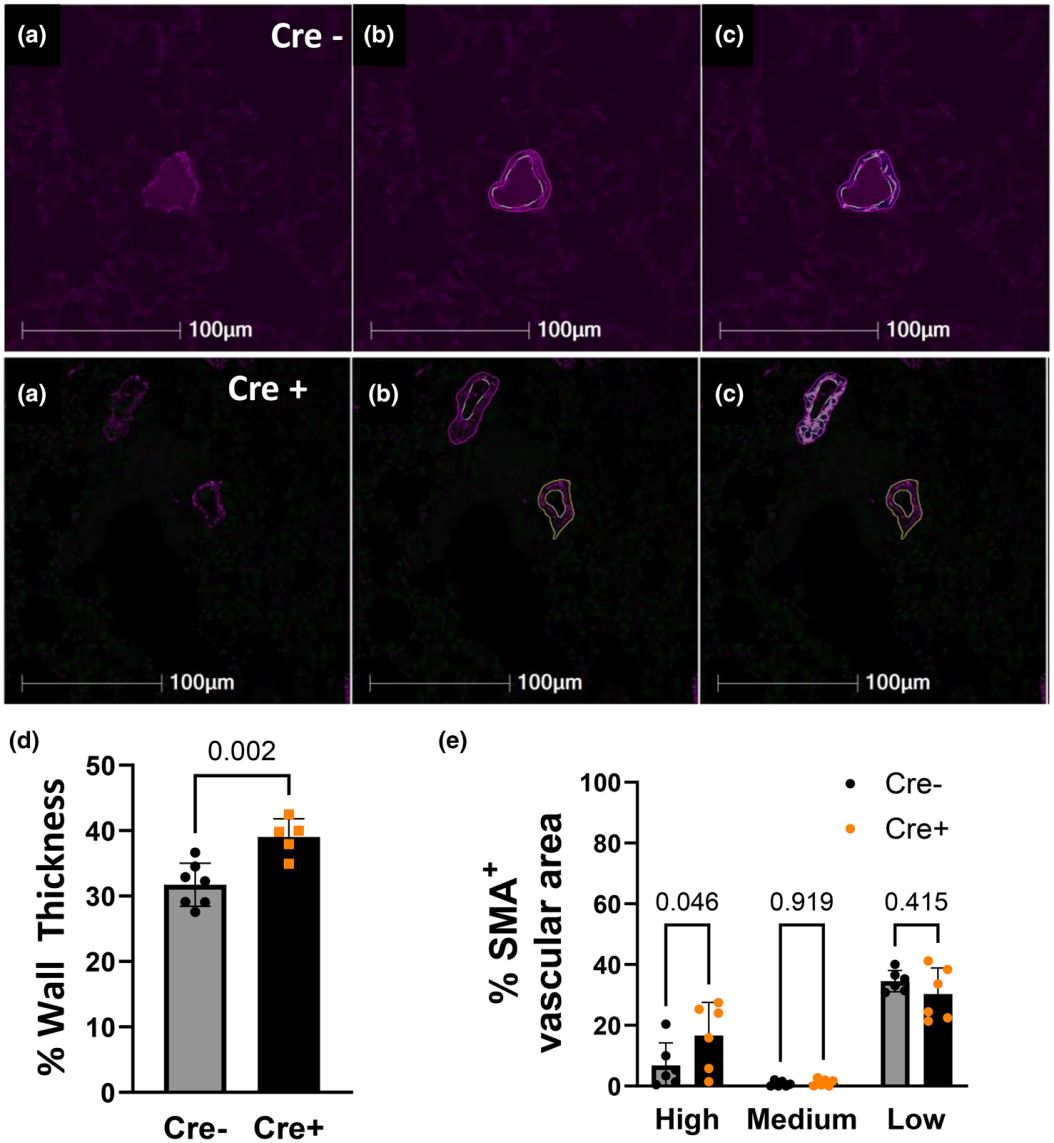
Pulmonary arterial remodeling develops in *Ift88* KO (Cre+) mice after 14 weeks of gene deletion. Immunofluorescence staining of lung sections was used to quantify pulmonary arterial wall thickness in arteries with an outer diameter of < 60 μm using HALO software (version 4.1, Indica Labs). (a) No mask, (b) inner and outer diameter selection, (c) mask based on smooth muscle α‐actin positive staining, (d) summary data of wall thickness, (e) summary data of % SMA+ vascular area. Approximately 10 arteries per animal were analyzed, and the averages were calculated per mouse. No sex‐based differences were detected. Data presented as mean ± SD. Unpaired *t*‐test. *n* = 7 and 5 mice, respectively.

Surprisingly, the average RVSP values of the control group of mice, which ranged from 20 to 30 mmHg, were higher than those previously reported by other groups and by us for healthy C57B6/J mice (Bierer et al., 2011; Ramiro‐Diaz et al., 2013; Thibault et al., 2010). Therefore, we performed a post hoc unsupervised clustering of the RVSP values (Figure 4b), revealing two clusters within the control group: one with RVSP values below 30 mmHg (5 mice) and the other with values above 30 mmHg (4 mice). No such clustering was present in the Cre + group, and all Cre + mice had RVSP values >30 mmHg. Based on these results, we separated the control group into two groups and compared RVSP values with those of the Cre + mice, showing that the Cre + mice had significantly higher RVSP than the group with RVSP values <30 mmHg (Figure 4c). The clustering of the values was not due to sex differences since RVSP did not correlate with sex. Furthermore, there was a significant sex effect on BW within the control group, with females having lower BW than males, as expected at that age; however, this sex difference was absent in the Cre + mice, where females had higher BW than control mice. At this time point, pulmonary arterial wall thickness was significantly elevated in Ift88 KO mice compared with control mice, together with expression levels of SMA, regardless of sex (Figure 5), suggesting that Ift88 KO mice have features of PH even after 14 weeks postgene deletion.

### 3.2 Potential Mechanisms Underlying PH Development in *Ift88* KO Mice

**FIGURE 6:**
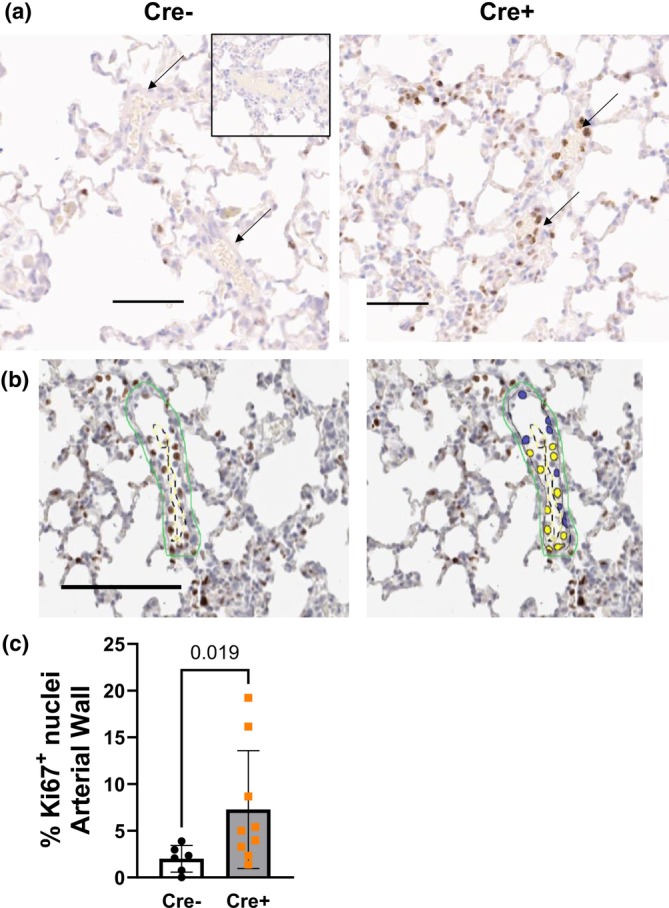
*Ift88* KO (Cre+) mice display increased pulmonary arterial cell proliferation 2 weeks after gene deletion. (a) Representative images of immunohistochemistry detection of Ki67+ nuclei in the arterial wall. Insert = no primary antibody control. (b) Digital amplification of a small artery from a Cre + mouse showing Ki67+ nuclei in both the lumen and media regions. Yellow mask = +, blue = −. (c) Summary of the percentage of proliferating cells within the walls of small pulmonary arteries in *Ift88* KO (Cre+) mice compared to control (Cre−) mice at 2 weeks post gene deletion. Data presented as mean ± SD. Mann–Whitney test. Arrows depict arterioles. *n* = 6 and 9 mice, respectively. The total number of cells counted per section ranged from 35 to 134, 5–6 arteries were selected per section per animal. Scale bar = 100 μm.

We (Bierer et al., 2011) and others (Garcia et al., 2022; Paddenberg et al., 2007) have previously demonstrated that cell proliferation within the pulmonary arterial wall precedes and declines before increases in pulmonary arterial wall thickness in a PH hypoxic model. To identify cellular processes contributing to PH development in Ift88 KO mice, we quantified cellular proliferation in the walls of small pulmonary arteries. Figure 6 shows that the % of Ki67^+^ nuclei was significantly higher in the wall of small pulmonary arteries from *Ift88* KO mice compared to controls after 2 weeks of *Ift88* gene deletion.

**FIGURE 7:**
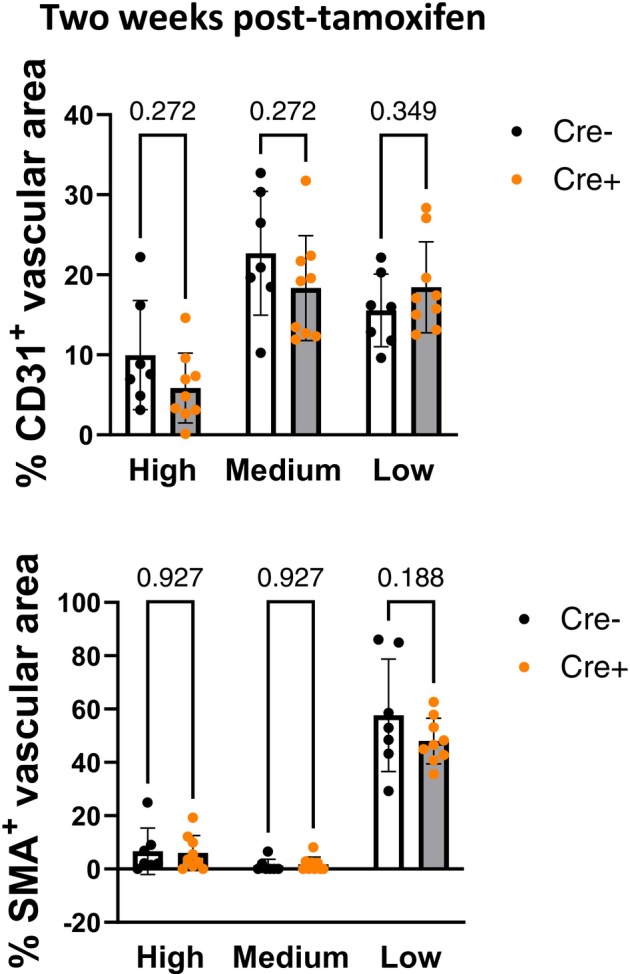
EndMT markers, CD31 and SMA, were not significantly altered in *Ift88* KO (Cre+) mice at 2 weeks post gene deletion. Immunohistochemistry analysis of CD31 and smooth muscle α‐actin (SMA) positive area with low, medium and high intensity within the walls of small pulmonary arteries in *Ift88* KO (Cre+) mice compared to controls. Data presented as mean ± SD. *n* = 7 and 9 mice, ~20 arteries/section/mouse. Multiple unpaired *t*‐tests.

Cellular proliferation was not associated with a change in the expression of EndMT markers, such as CD31 or SMA, at the early time point (Figure 7).

**FIGURE 8:**
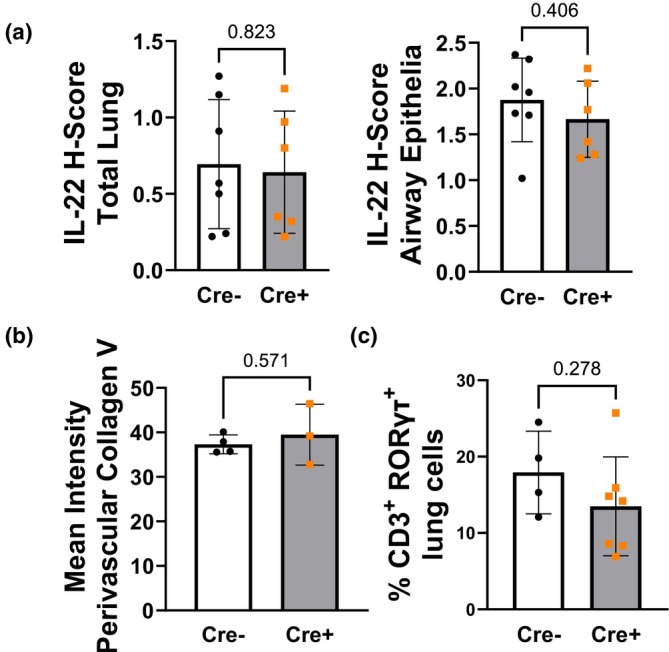
Lung inflammation markers are not elevated in *Ift88* KO (Cre+) mice 2 weeks after gene deletion. (a) Quantification of IL‐22 immunoreactivity in both whole lung tissue and airway epithelial regions in *Ift88* KO and control (Cre−) mice after 2 weeks posttamoxifen administration. *n* = 7 and 6 mice. (b) Summary data (*n* = 4 and 3 mice, 6 arteries/per mouse). (c) Percentage of Th17 cells (CD3+ RORγ+) in lung sections of *Ift88* KO and control mice (*n* = 4 and 7 mice). Data presented as mean ± SD. Unpaired *t*‐test.

Because IL‐22 concentration was increased in the bronchoalveolar lavage fluid of *Ift88* KO mice (14 weeks post‐tamoxifen) compared to controls (Nava et al., 2022), and IL‐22 has been shown to play a role in PH pathogenesis (Bansal et al., 2013; Crnkovic et al., 2016), we assessed the expression levels of IL‐22 in lung sections. Figure 8a shows that IL‐22 immune reactivity was not different between *Ift88* KO and control mice, either when quantified in the whole lung or airway epithelia after 2 weeks post‐*Ift88* gene deletion. Furthermore, we stained for collagen V, a self‐antigen that triggers T_H_17 cell‐dependent collagen V immune reactivity and has been demonstrated to be increased in the pulmonary perivascular region following hypobaric hypoxia in mice (Sheak et al., 2020). Figure 8b,c shows that the collagen V perivascular immune reactivity was not significantly different between groups and was low, consistent with our previous findings in lungs from normoxic wild‐type mice (Sheak et al., 2020). Figure 8d shows no difference in the % of T_H_17 cells in lung sections of *Ift88* KO compared to control mice. These results suggest that lung inflammation was not responsible for the PH present in *Ift88* KO mice 2 weeks after gene deletion.

## 4 DISCUSSION

This study reports the novel finding that features of mild PH, such as elevated RVSP, pulmonary arterial wall cell proliferation and increased wall thickness, are present in adult mice with inducible global deletion of *Ift88*. The elevated RVSP and cell proliferation occurred before the development of ciliopathy or any other comorbidities previously reported in this mouse strain (Gilley et al., 2014; Nava et al., 2022). Although we detected an elevated RVSP, a surrogate for pulmonary artery pressure, in *Ift88* KO mice 2 weeks postgene deletion, we did not observe RV hypertrophy. Interestingly, this is not the only animal model in which PH develops without a concomitant increase in RV weight. We reported a similar finding in *Sod1* KO mice (Ramiro‐Diaz et al., 2013), and others demonstrated PH development without a concomitant rise in RV weight in nitric oxide synthase 3‐deficient mice (Steudel et al., 1998) and young transgenic Re2 rats (DeMarco et al., 2008). The mechanism of this dissociation remains unclear.

Our data suggest that a potential mechanism by which *Ift88* deletion causes PH is increased proliferation of arterial wall cells. This is consistent with a report showing that IFT88 siRNA knockdown in human umbilical vein, human coronary artery and human pulmonary artery ECs increases cell proliferation and results in a simultaneous loss of primary cilia (Singh et al., 2020). However, we did not observe a predominance of proliferating cells in either the lumen or the media regions. The same study also showed that *IFT88* knockdown induced EndMT in human ECs, as evidenced by decreased CD31 and increased SMA expression, among other markers (Singh et al., 2020). However, we did not detect changes in CD31 or SMA expression in pulmonary arteries from *Ift88* KO mice at the early time point when we observed elevated Ki‐67 expression. SMA expression was higher in the media layer of the pulmonary arteries at a later time point, coinciding with elevated pulmonary arterial wall thickness in *Ift88* KO mice, suggesting pulmonary arterial remodeling, another hallmark of PH (de Frutos et al., 2009, 2007). A limitation of the study is that SMA expression levels cannot be compared across the two time points because the assessment was conducted using two different methods.

The RVSP data within the control group (Cre−) in the 14‐week time point appeared to cluster into two groups, >30 and < 30 mmHg, whereas such clustering was not observed in Ift88 KO mice or in the two‐week posttamoxifen mice. The clustering was determined post hoc and appears to be independent of sex differences since RVSP did not correlate with sex. The clustering in the control group cannot be attributed to differences in age or tamoxifen administration, since the mice were age‐matched and all received tamoxifen. A possible explanation is that differences in weight determined the RVSP, since RVSP significantly correlated with BW. However, further studies are needed to establish the cause of these results. Interestingly, all Ift88 KO mice had RVSP that were above 30 mmHg. Typical RVSP baseline values for healthy mice on a C57B6/J background range from 20 to 30 mmHg (Bierer et al., 2011; Ramiro‐Diaz et al., 2013; Thibault et al., 2010), suggesting that Ift88 KO mice might have PH even at the later time point. Although the data are inconclusive, the hypothesis is supported by the presence of pulmonary arterial remodeling and increased SMA expression in the same mice.

At the early time point, we did not detect evidence of lung inflammation or EndMT that could explain the signs of PH observed in Ift88 KO mice, as the expression of IL‐22, collagen V and the percentage of T_H_17 cells in the lungs of these mice did not differ from those of controls. We focused on these molecules and cells because we previously demonstrated that 3 months after deleting Ift88, mice exhibit lung airway and perivascular inflammation, elevations in IL‐22 and IL‐6 in BALF and a significant decrease in total lung Tregs (Nava et al., 2022). Furthermore, in wild‐type mice, PH develops, in part, due to immunologic dysregulation involving a T_H_17/Treg imbalance (Lantz et al., 2024), which contributes to vascular remodeling of the pulmonary vasculature (Cuttica et al., 2011; Funk‐Hilsdorf et al., 2022; Huertas et al., 2016; Maston et al., 2017). Upregulation of the self‐antigen collagen V contributes to the T_H_17 cell‐mediated inflammation in PH (Sheak et al., 2020). Additionally, IL‐22 has been shown to contribute to vascular endothelial dysfunction and inflammation, and inflammatory cytokines promote EndMT (Gong et al., 2017). Furthermore, inflammatory cytokines cause cilia elongation in ECs from healthy subjects. In contrast, cilia are larger at baseline and respond less to cytokines in cells from patients with PAH, a phenotype indicative of endothelial dysfunction (Dummer et al., 2018). However, our data suggest that inflammation following *Ift88* deletion takes more than 2 weeks to develop and may not be the underlying cause of the early signs of PH in these mice. However, we cannot exclude a contribution of inflammation at later stages, even if it does not appear to drive the initial rise in RVSP.

Because this is a global Ift88 KO model (Gilley et al., 2014; Nava et al., 2022), we cannot assign the PH phenotype specifically to endothelial or smooth muscle Ift88 gene deletion. In addition, we cannot discard the possibility that tamoxifen administration, global Ift88 deletion and the associated weight gain and food restriction may have influenced cardiovascular physiology in these mice. The observed correlation between RVSP and body weight at the 14‐week time point is worth noting as a limitation.

## AUTHOR CONTRIBUTIONS

S.M.G. data curation, formal analysis, investigation, methodology and review and editing of the manuscript. B.J.L. data curation, formal analysis, investigation, methodology and review and editing of the manuscript. H.J.W. data curation, formal analysis and review and editing of the manuscript. D.T.J. data curation, formal analysis, investigation, methodology and review and editing of the manuscript. R.A.‐G. data curation, formal analysis and review and editing of the manuscript. T.A.H. data curation, formal analysis, methodology and review and editing of the manuscript. S.G. data curation, methodology and review and editing of the manuscript. T.W. conceptualization, funding acquisition, investigation, methodology and review and editing of the manuscript. T.F.B. conceptualization, funding acquisition, investigation, methodology, resources and review and editing of the manuscript. O.C.H. data curation, formal analysis, methodology and review and editing of the manuscript. L.V.G.B. conceptualization, data curation, formal analysis, funding acquisition, investigation, methodology, project administration, resources, supervision, validation, visualization, original draft and review and editing of the manuscript.

## FUNDING INFORMATION

The author(s) declare that financial support was received for the research, authorship and/or publication of this article. The National Institutes of Health T32 HL07736 grant supported BJL's tuition and stipend. The American Heart Association (Transformational Award 967325) funding supported a portion of LBG's salary. The National Institutes of Health R01HL166740 supported a portion of LGB's and BJL's salaries. This research was partially funded by the UNM Comprehensive Cancer Center Support Grant NCI P30CA118100 through the use of the Human Tissue Repository Shared Resource.

## CONFLICT OF INTEREST STATEMENT

The authors declare no conflicts of interest.

## Data Availability

The data that support the findings of this study are available from the corresponding author upon reasonable request.
